# Bortezomib suppresses acute myelogenous leukaemia stem‐like KG‐1a cells via NF‐κB inhibition and the induction of oxidative stress

**DOI:** 10.1111/jcmm.18333

**Published:** 2024-04-23

**Authors:** Rafaela G. A. Costa, Maiara de S. Oliveira, Ana Carolina B. da C. Rodrigues, Suellen L. R. Silva, Ingrid R. S. B. Dias, Milena B. P. Soares, Ludmila de Faro Valverde, Clarissa Araujo Gurgel Rocha, Rosane Borges Dias, Daniel P. Bezerra

**Affiliations:** ^1^ Gonçalo Moniz Institute Oswaldo Cruz Foundation (IGM‐FIOCRUZ/BA) Salvador Bahia Brazil; ^2^ SENAI Institute for Innovation in Advanced Health Systems SENAI CIMATEC Salvador Bahia Brazil; ^3^ Department of Propaedeutics, Faculty of Dentistry Federal University of Bahia (UFBA) Salvador Bahia Brazil; ^4^ Center for Biotechnology and Cell Therapy D'Or Institute for Research and Education (IDOR) Salvador Bahia Brazil

**Keywords:** AML, bortezomib, leukaemic stem cells, NF‐κB, oxidative stress

## Abstract

Acute myelogenous leukaemia (AML) originates and is maintained by leukaemic stem cells (LSCs) that are inherently resistant to antiproliferative therapies, indicating that a critical strategy for overcoming chemoresistance in AML therapy is to eradicate LSCs. In this work, we investigated the anti‐AML activity of bortezomib (BTZ), emphasizing its anti‐LSC potential, using KG‐1a cells, an AML cell line with stem‐like properties. BTZ presented potent cytotoxicity to both solid and haematological malignancy cells and reduced the stem‐like features of KG‐1a cells, as observed by the reduction in CD34‐ and CD123‐positive cells. A reduction in NF‐κB p65 nuclear staining was observed in BTZ‐treated KG‐1a cells, in addition to upregulation of the NF‐κB inhibitor gene *NFΚBIB*. BTZ‐induced DNA fragmentation, nuclear condensation, cell shrinkage and loss of transmembrane mitochondrial potential along with an increase in active caspase‐3 and cleaved PARP‐(Asp 214) level in KG‐1a cells. Furthermore, BTZ‐induced cell death was partially prevented by pretreatment with the pancaspase inhibitor Z‐VAD‐(OMe)‐FMK, indicating that BTZ induces caspase‐mediated apoptosis. BTZ also increased mitochondrial superoxide levels in KG‐1a cells, and BTZ‐induced apoptosis was partially prevented by pretreatment with the antioxidant *N*‐acetylcysteine, indicating that BTZ induces oxidative stress‐mediated apoptosis in KG‐1a cells. At a dosage of 0.1 mg/kg every other day for 2 weeks, BTZ significantly reduced the percentage of hCD45‐positive cells in the bone marrow and peripheral blood of NSG mice engrafted with KG‐1a cells with tolerable toxicity. Taken together, these data indicate that the anti‐LSC potential of BTZ appears to be an important strategy for AML treatment.

## 1 INTRODUCTION

Acute myelogenous leukaemia (AML) is a malignant haematological disease characterized by the accumulation of immature blasts in the bone marrow and blood, resulting in ineffective haematopoiesis and bone marrow failure. AML is a highly heterogeneous disease that represents a major clinical challenge due to its variable prognosis.^1^, ^2^, ^3^, ^4^ In the United States of America, the overall 5‐year survival rate of patients with AML was only 28% from 2010 to 2017.^5^ Although some pharmacological targets have recently been identified for anti‐AML therapy, new therapeutic drugs are needed mainly for patients with pharmacotherapeutic resistance.

AML originates from leukaemia‐initiating cells that have stem cell properties and are called leukaemic stem cells (LSCs). These cells are thought to have inherent resistance to antiproliferative therapies, indicating that a critical strategy for overcoming chemoresistance in AML therapy is eradicating LSCs.^6^ Multiple cell signalling pathways, as well as the NF‐ĸB pathway, have been shown to be important targets for suppressing AML LSCs.^7^ In addition, the induction of oxidative stress has also been indicated as a target to eliminate AML LSCs.^8^

**FIGURE 1:**
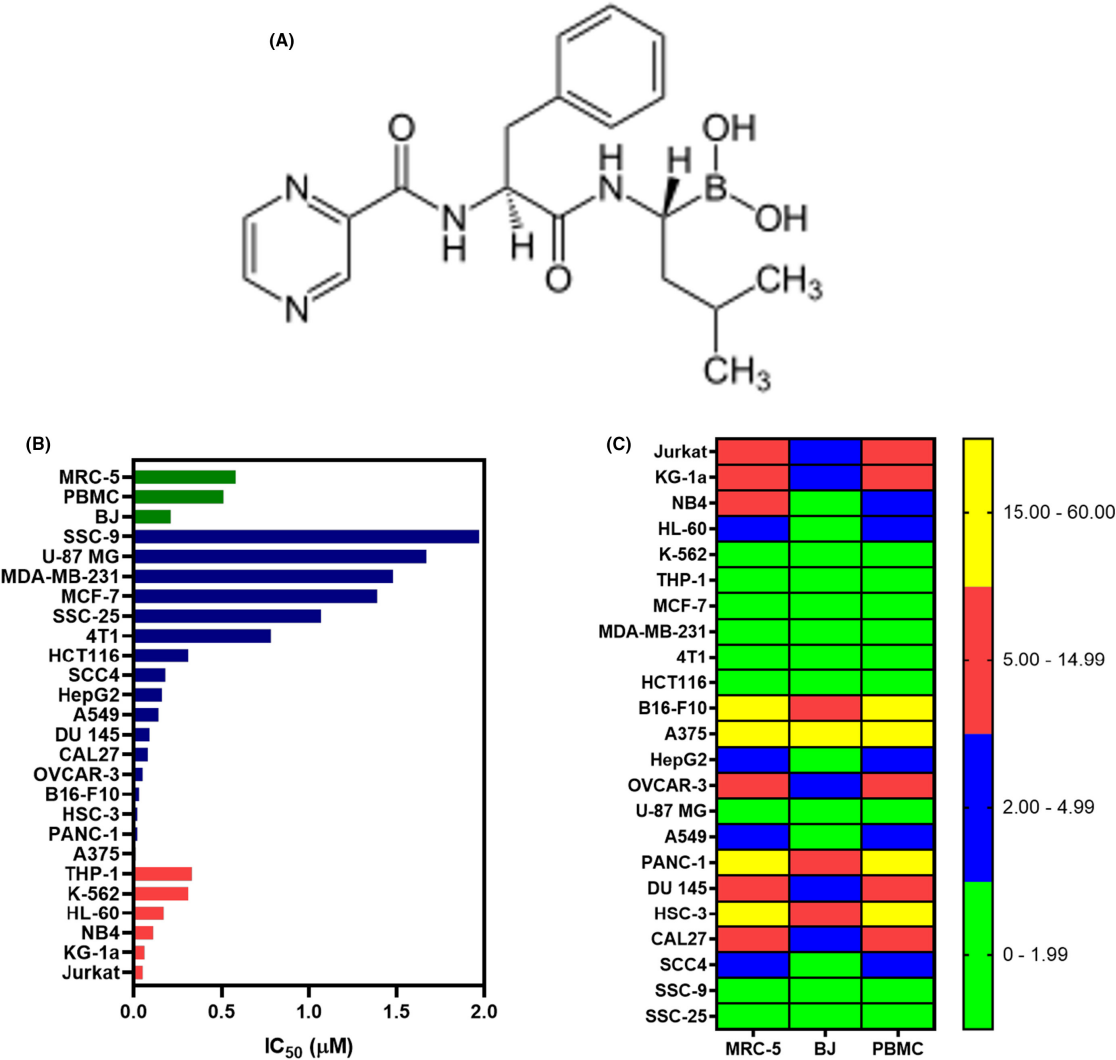
BTZ induces potent and selective cytotoxicity to both solid and haematological malignant cells. (A) Chemical structure of BTZ. (B) IC_50_ values showing the cytotoxicity of BTZ against haematological (red bars) and solid cancers (blue bars), as well as against noncancerous cells (green bars). (C) Heatmap of selectivity indices (SI) calculated for BTZ. The SI was calculated using the following formula: SI = IC_50_ [noncancerous cells]/IC_50_ [cancer cells].

Bortezomib (BTZ, Figure 1A) is a FDA‐approved treatment of multiple myeloma, and mantle cells lymphoma that act as a selective and reversible inhibitor of the 26S proteasome.^9^ In addition, BTZ may be effective in treating other haematological neoplastic diseases, such as T‐cell acute lymphoblastic leukaemia,^10^ lymphoma^10^ and AML.^11^ BTZ has been reported to inhibit the stemness features of AML, including AML with lysine [K]‐methyltransferase 2A (KMT2A)/mixed‐lineage leukaemia 1 (KMT2A/MLL) rearrangements, a type of aggressive AML, via NF‐ĸB‐dependent inhibition of CDK6.^12^, ^13^

In this work, we investigated the anti‐AML activity of BTZ, emphasizing its anti‐LSC potential, using KG‐1a cells, an AML cell line with stem‐like properties.^14^ We found that BTZ reduces the number of AML stem‐like cells via NF‐κB inhibition and the induction of oxidative stress.

## 2 MATERIALS AND METHODS

### 2.1 Drug

BTZ was obtained commercially (Cayman Chemical; Ann Arbor, MI, USA).

### 2.2 In vitro assays

#### 2.2.1 Cells

A total of 23 cancer cell lines, two noncancerous cell lines, one noncancerous primary cell and one mutant cell line and its parental control cell line were used in the study and are detailed in Table S1. The cells were cultured in flasks at 37°C in 5% CO_2_ and were replicated every 3–4 days to maintain exponential cellular growth, following the instructions of the ATCC animal cell culture guidelines. A 0.25% trypsin EDTA solution (Sigma–Aldrich Co., Saint Louis, MO, USA) was used to remove adherent cells. All cell lines tested negative for mycoplasma, as evaluated by a mycoplasma stain kit (Sigma Aldrich Co.). All experiments were performed with cells at fewer than 50 passages.

The concentration of viable cells was determined by the trypan blue exclusion method. For that, 90 μL of the cell suspension was mixed with 10 μL of trypan blue (0.4%), and a haemocytometer was used to count viable (unstained cells) and nonviable (trypan blue‐stained cells) cells using a light microscope.

#### 2.2.2 Alamar blue assay

Cell viability was quantified by the Alamar blue assay.^15^ Briefly, the cells were plated in 96‐well culture plates (30,000 cells/well for suspension cells or 7000 cells/well for adherent cells) and kept at 37°C in a 5% CO_2_ atmosphere. BTZ was added to each well in duplicate and incubated for 72 h. Doxorubicin (purity ≥95%, Laboratory IMA S.A.I.C., Buenos Aires, Argentina) was used as a positive control. Four hours before the end of the incubation period (or 24 h for PBMCs), resazurin was added to each well at a final concentration of 3 μM. The absorbance values at 570 nm and 600 nm were evaluated using a SpectraMax 190 Microplate Reader (Molecular Devices, Sunnyvale, CA, USA).

#### 2.2.3 Immunophenotyping assay

Phenotyping of KG‐1a cells was performed using primary antibodies against CD11b, CD13, CD33, CD34, CD38 and CD123 conjugated with specific fluorochromes (Table S2). For that, the cells were rinsed with incubation buffer (0.5% bovine serum albumin in PBS) and incubated with antibodies for 1 h at room temperature. Then, the cells were rinsed with PBS, stained with YO‐PRO‐1 (Sigma–Aldrich Co.) and analysed by flow cytometry. A BD LSRFortessa cytometer using BD FACSDiva Software (BD Biosciences, San Jose, CA, USA) and FlowJo Software 10 (FlowJo LCC, Ashland, OR, USA) was used. At least 30,000 events were analysed per sample. Cell doublets and debris were excluded from the analyses.

#### 2.2.4 Immunofluorescence by confocal microscopy

The localization of NF‐κB p65 was investigated by confocal microscopy. Briefly, cells were washed twice with PBS, plated as droplets (5 μL) on coverslips, permeabilized with Triton X‐100 (0.5%), treated with RNase (10 μg/mL), washed with PBS and incubated overnight with an anti‐NF‐κB p65 antibody (Table S2 contains antibody details). The following day, the cells were washed with PBS and mounted with Fluoromount‐G (Invitrogen, Thermo Fisher Scientific) containing DAPI. The cells were photographed using a Leica TCS SP8 confocal microscope (Leica Microsystems, Wetzlar, HE, Germany).

#### 2.2.5 qPCR array

Total RNA was extracted using the RNeasy Plus Mini Kit (Qiagen; Hilden, Germany) according to the manufacturer's instructions. RNA purity was examined and quantified using a NanoDrop® 1000 spectrophotometer (Thermo Fisher Scientific, Waltham, MA, USA). RNA reverse transcription was evaluated using the Superscript VILO™ kit (Invitrogen Corporation; Waltham, MA, USA). qPCR analysis of gene expression was performed on a TaqMan® Array Plate 96 plus fast (#4413256, Applied Biosystems™, Foster City, CA, USA) on an ABI ViiA7 system (Applied Biosystems™). The PCR cycling conditions were 50°C for 2 min and 95°C for 10 min, followed by 40 cycles of 95°C for 15 s and 60°C for 1 min. Relative quantification (RQ) of mRNA expression was calculated using the 2^−ΔΔCT^ method^16^ on Gene Expression Suite™ software (Applied Biosystems™). Cells treated with 0.2% DMSO (negative control) were used as calibrators. The geometric mean RQs of the three reference genes GUSB, HPRT1 and GAPDH were considered for data normalization. All experiments were performed under DNase/RNase‐free conditions. A gene was upregulated if its RQ ≥2. Similarly, genes were downregulated when RQ ≤0.5.

#### 2.2.6 Internucleosomal DNA fragmentation and cell cycle evaluation

Internucleosomal DNA fragmentation and cell cycle progression were assessed by flow cytometry, which detected DNA content with propidium iodide (PI).^17^ The cells were stained with a solution containing 0.1% Triton X‐100, 2 μg/mL PI, 0.1% sodium citrate, and 100 μg/mL RNase (all from Sigma Aldrich Co.). The cells were analysed by flow cytometry after 15 min of incubation in the dark, as described above. At least 10,000 events were examined per sample.

#### 2.2.7 Apoptosis assays

Apoptotic cells were detected by annexin V‐FITC/PI (FITC Annexin V Apoptosis Detection Kit I, BD Biosciences) or YO‐PRO‐1/PI (Sigma–Aldrich Co.) according to the manufacturer's instructions. The cells were analysed by flow cytometry, as described above. At least 10,000 events were analysed per sample. The antioxidant *N*‐acetylcysteine (NAC) and the pancaspase inhibitor Z‐VAD(OMe)‐FMK were used in the functional assays.

Mitochondrial transmembrane potentials were evaluated using cells stained with rhodamine 123.^18^ After 24 h of treatment, the cells were stained with 1 μg/mL rhodamine 123 (Sigma–Aldrich Co.) and incubated for 15 min at 37°C in the dark. After that, the cells were rinsed and analysed by flow cytometry, as described above. At least 10,000 events were analysed per sample.

The levels of active caspase‐3 and cleaved PARP (Asp 214) were quantified using primary antibodies conjugated with specific fluorochromes (Table S2) following a protocol for intracellular staining of cells. The cells were collected and resuspended in 0.5–1 mL of 4% formaldehyde for 10 min at 37°C. The tube was then placed on ice for 1 min. Cells were permeabilized on ice for 30 min by slowly adding ice‐cold 100% methanol to prechilled cells with gentle vortexing until the final concentration of methanol reached 90%. After washing with an incubation buffer (0.5% bovine serum albumin in PBS), primary antibodies were added and incubated for 1 h at room temperature. Then, the cells were rinsed with PBS and analysed by flow cytometry, as described above. At least 10,000 events were analysed per sample.

#### 2.2.8 Mitochondrial superoxide levels

Mitochondrial superoxide levels were quantified using MitoSOX™ Red reagent (Thermo Fisher Scientific, Waltham, MA, USA), and analysis was performed according to the manufacturer's instructions. The cells were analysed by flow cytometry, as described above. At least 10,000 events were analysed per sample.

### 2.3 In vivo assay

#### 2.3.1 Animals

A total of 12 specific pathogen‐free NOD. Cg‐Prkdc^scid^ Il2rg^tm1Wjl^/SzJ (NSG) mice (male and female, 20–25 g) were obtained and kept by FIOCRUZ‐BA animal facilities (Salvador, Bahia, Brazil). The experimental protocol was approved by a local animal ethics committee (#16/2018). All animals were fed a standard pellet diet (food and water available ad libitum) and housed in an artificially lit room (12 h dark/light cycle).

#### 2.3.2 Xenotransplantation of leukaemia cells

One day before receiving the KG‐1a cells, the animals were treated with 25 mg/kg busulfan (Sigma–Aldrich Co.) to allow high bone marrow engraftment. The next day, the animals were injected with 10^6^ cells per mouse via the tail vein. Every day, all animals were observed for symptoms of weight loss or lethargy. Engraftment was verified in peripheral blood after 2 weeks using both PE‐conjugated antihuman CD45 (hCD45) and FITC‐conjugated anti‐mouse CD45 (mCD45) antibodies (Table S2) by flow cytometry, as described above.

Animals were randomly separated into two groups (*n* = 6) after engraftment confirmation: group 1, treated with vehicle (5% DMSO, negative control); and group 2, treated with BTZ at a dose of 0.1 mg/kg. The animals received intraperitoneal treatments every other day for 2 weeks. The animals were then euthanized with an anaesthetic overdose (thiopental, 100 mg/kg), and cells were collected from the spleen, peripheral blood and bone marrow. These cells were stained with hCD45 and mCD45 antibodies and analysed by flow cytometry, as described above. At least 30,000 events were analysed per sample.

#### 2.3.3 Toxicology analysis

In addition, the kidneys, lungs, hearts and livers were removed for toxicological analysis. These organs were checked for colour change, gross lesion development and/or bleeding before being fixed in 4% formaldehyde, dehydrated through a graded alcohol series, rinsed in xylene and wrapped in paraffin wax. Tissues were cut into 5 μm thick slices, stained with haematoxylin–eosin and/or periodic acid‐Schiff (liver and kidney), and examined histologically under a light microscope.

### 2.4 Statistical analysis

The data are shown as the mean ± S.E.M. or as IC_50_ values with a 95% confidence interval from at least three independent repetitions (done in duplicate). The selectivity indices (SI) were calculated using the following formula: SI = IC_50_ [noncancerous cells]/IC_50_ [cancer cells]. Two‐tailed unpaired Student's *t*‐test (*p* < 0.05) was used to compare data between two groups, and one‐way analysis of variance (ANOVA) followed by Dunnett's multiple comparisons test (*p* < 0.05) was used to compare data among three or more groups. All the statistical analyses were performed with GraphPad Prism (Intuitive Software for Science; San Diego, CA, USA).

## 3 RESULTS

### 3.1 BTZ has potent cytotoxic effects on both solid and haematological malignant cells

The cytotoxic effect of BTZ was evaluated in a panel of 17 solid cancer cell lines (MDA‐MB‐231, MCF‐7, 4T1, HCT116, B16‐F10, A‐375, HepG2, OVCAR‐3, U‐87 MG, A549, PANC‐1, DU145, HSC‐3, CAL27, SSC‐4, SCC‐9 and SSC‐25), six haematological cancer cell lines (Jurkat, KG‐1a, NB4, HL‐60, K‐562 and THP‐1) and three noncancerous cell lines (MRC‐5, BJ and PBMCs) by the Alamar blue assay after 72 h of incubation. Figure 1B and Table S3 present the results. In solid cancer cells, BTZ showed IC_50_ values ranging from 0.01 to 1.83 μM for melanoma A375 cells and oral squamous cell carcinoma SCC‐9 cells, respectively. In haematological malignant cells, BTZ had IC_50_ values ranging from 0.05 to 0.33 μM for the T‐cell lymphoid leukaemia Jurkat and monocytic leukaemia THP‐1 cell lines, respectively. Doxorubicin, which was used as a positive control, had IC_50_ values ranging from 0.04 to 4.36 μM for T‐cell lymphoblastic leukaemia Jurkat cells and pancreatic cancer PANC‐1 cells, respectively.

For noncancerous cell lines, BTZ had IC_50_ values of 0.58, 0.21 and 0.51 μM for lung fibroblast MRC‐5 cells, foreskin fibroblast BJ cells and PBMCs, respectively. The selectivity indices were calculated and are shown in Figure 1C and Table S4. The selectivity of BTZ for normal PBMCs compared with that for AML cells was greater than 2‐fold for Jurkat (10.2), KG‐1a (8.5), NB4 (4.6) and HL‐60 (3.0) cells, indicating good selectivity. Doxorubicin had IC_50_ values of 1.44, 2.65 and 1.33 μM for lung fibroblast MRC‐5 cells, foreskin fibroblast BJ cells and PBMCs, respectively.

### 3.2 BTZ reduces the stem‐like features of KG‐1a cells

KG‐1a cells are an AML cell line with stem‐like properties^14^ and were selected for subsequent experiments. The anti‐AML activity of BTZ in KG‐1a cells was evaluated at concentrations of 0.5, 1 and 2 μM. Next, a trypan blue exclusion assay was used to confirm the effect of BTZ on KG‐1a cells after 12, 24, 48 and 72 h of incubation. BTZ reduced the number of viable KG‐1a cells in a time‐dependent manner (Figure S1).

**FIGURE 2:**
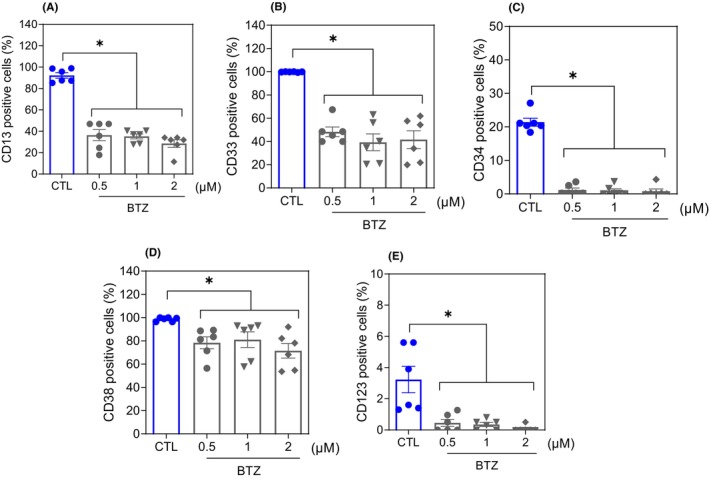
BTZ suppresses the stem‐like features of KG‐1a cells. Immunophenotypic analysis of the myeloid lineage markers CD13 (A) and CD33 (B) and the AML stem/progenitor markers CD34 (C), CD38 (D) and CD123 (E) in BTZ‐treated KG‐1a cells after 48 h of incubation. The vehicle (0.2% DMSO) was used as a negative control (CTL). The data are shown as the mean ± S.E.M. of three independent experiments carried out in duplicate. *p < 0.05 compared to CTL by one‐way ANOVA followed by Dunnett's multiple comparisons test.

The effect of 0.5, 1 and 2 μM BTZ on the stem‐like features of AML KG‐1a cells was evaluated by flow cytometric immunophenotyping using the myeloid lineage markers CD13^19^ and CD33^20^ and the AML stem/progenitor markers CD34,^21^ CD38^21^ and CD123^22^ after 48 h of incubation (Figure 2). Treatment with BTZ reduced the expression of both myeloid lineage and AML stem/progenitor markers, indicating that BTZ can inhibit the stemness features of KG‐1a cells. CD34 and CD123 are specific markers of LSCs, while CD38 can mark proliferative progenitor cells but not LSCs. A reduction in these markers indicates stem/progenitor cell suppression. The expression of CD11b,^23^ an early myeloid differentiation marker, was also evaluated after 24 h of incubation with BTZ at a concentration of 2 μM (Figure S2). However, no change was observed.

### 3.3 BTZ inhibits NF‐κB signalling and downregulates genes related to stemness properties in KG‐1a cells

**FIGURE 3:**
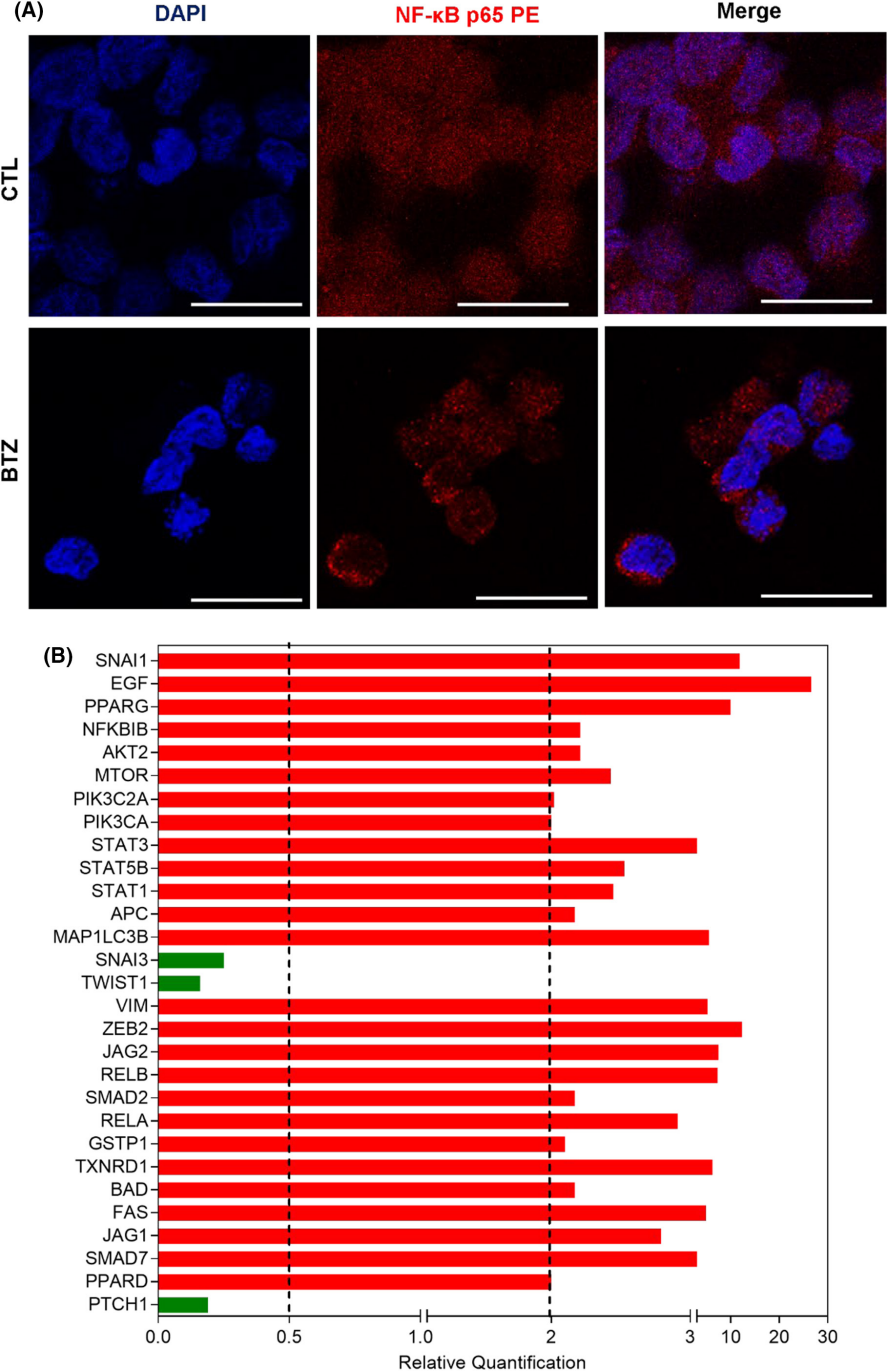
BTZ reduces NF‐κB signalling and downregulates genes related to stemness properties in KG‐1a cells. (A) Representative immunofluorescence images of NF‐κB p65 in KG‐1a cells after 4 h of incubation with 2 μM BTZ. Scale bar = 25 μm. (B) Up‐ and down‐regulated genes in KG‐1a cells after 12 h of treatment with 2 μM BTZ. Genes that displayed RQ ≥2 (red bars) were upregulated, and RQ ≤0.5 (green bars) were downregulated.

As BTZ is a known NF‐κB inhibitor,^24^ we evaluated whether it could inhibit NF‐κB signalling in KG‐1a cells. A reduction in NF‐κB p65 nuclear staining was observed in BTZ‐treated KG‐1a cells after 4 h of incubation (Figure 3A), indicating interference with this cell signalling pathway.

Furthermore, the effect of BTZ on the expression of a panel of genes related to stemness properties was investigated by a qPCR array (Figure 3B and Table S5). Among the 92 genes analysed, BTZ upregulated the expression of 26 genes and downregulated the expression of three genes. BTZ upregulated the NF‐κB inhibitor gene *NFΚBIB* (RQ = 2.21) and the proapoptotic genes *BAD* (RQ = 2.17) and *FAS* (RQ = 4.93). The hedgehog signalling receptor gene *PTCH1* (RQ = 0.19) and the epithelial–mesenchymal transition marker genes *SNAI3* (RQ = 0.25) and *TWIST1* (RQ = 0.16) were downregulated in KG‐1a cells.

### 3.4 BTZ induces caspase‐mediated apoptosis in KG‐1a cells

**FIGURE 4:**
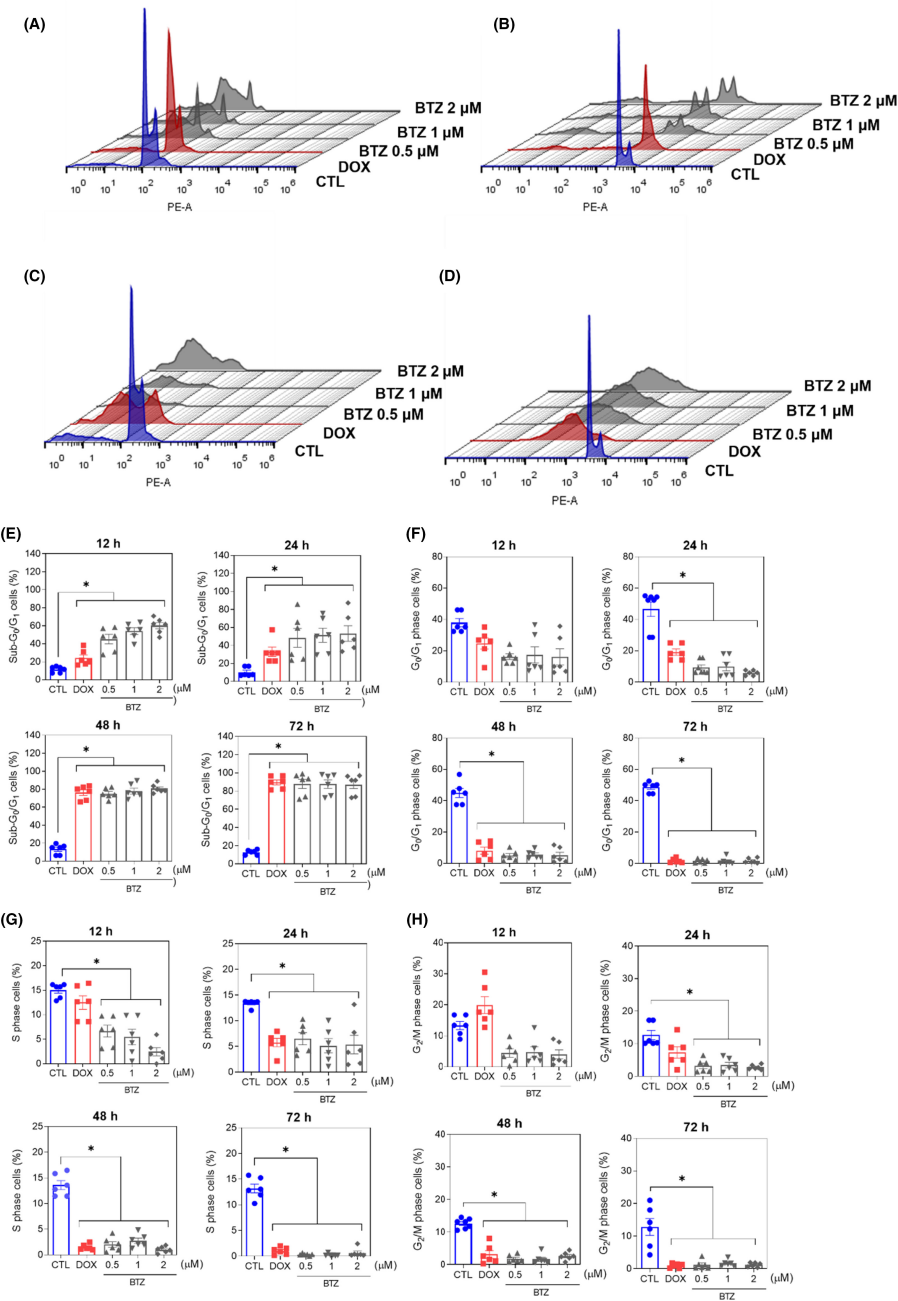
BTZ affects cell cycle progression in KG‐1a cells. Representative histograms after (A) 12, (B) 24, (C) 48 and (D) 72 h of treatment. Percentages of cells in (E) sub‐G_0_/G_1_, (F) G_0_/G_1_, (G) S and (H) G_2_/M after different incubation periods with BTZ. Vehicle (0.2% DMSO) was used as a negative control (CTL), and doxorubicin (DOX, 1 μM) was used as a positive control. The data are shown as the mean ± S.E.M. of three independent experiments carried out in duplicate. *p < 0.05 compared with CTL by one‐way ANOVA followed by Dunnett's multiple comparisons test.

The mechanism underlying BTZ‐induced cell death was evaluated. First, cell cycle progression and internucleosomal DNA fragmentation were investigated in BTZ‐treated KG‐1a cells. All cells with subdiploid DNA content (sub‐G_0_/G_1_ subpopulation) were considered to have fragmented DNA. BTZ‐induced DNA fragmentation in a time‐ and concentration‐dependent manner (Figure 4). At concentrations of 0.5, 1 and 2 μM, BTZ‐induced DNA fragmentation by 45.3%, 54.2% and 60.2%, respectively, after 12 h of incubation (in contrast to the 11.5% detected in the control); by 48.4%, 51.3% and 53.3%, respectively, after 24 h of incubation (in comparison to the 10.5% found in the control); by 75.3%, 78.0% and 80.7%, respectively, after 48 h of incubation (in comparison to the 13.3% in the control); and by 87.9%, 87.8% and 87.3%, respectively, after 72 h of incubation (in comparison to the 12.8% in the control). The phases of the cell cycle (G_0_/G_1_, S and G_2_/M) were reduced proportionally. Doxorubicin was used as a positive control, and DNA fragmentation was induced.

**FIGURE 5:**
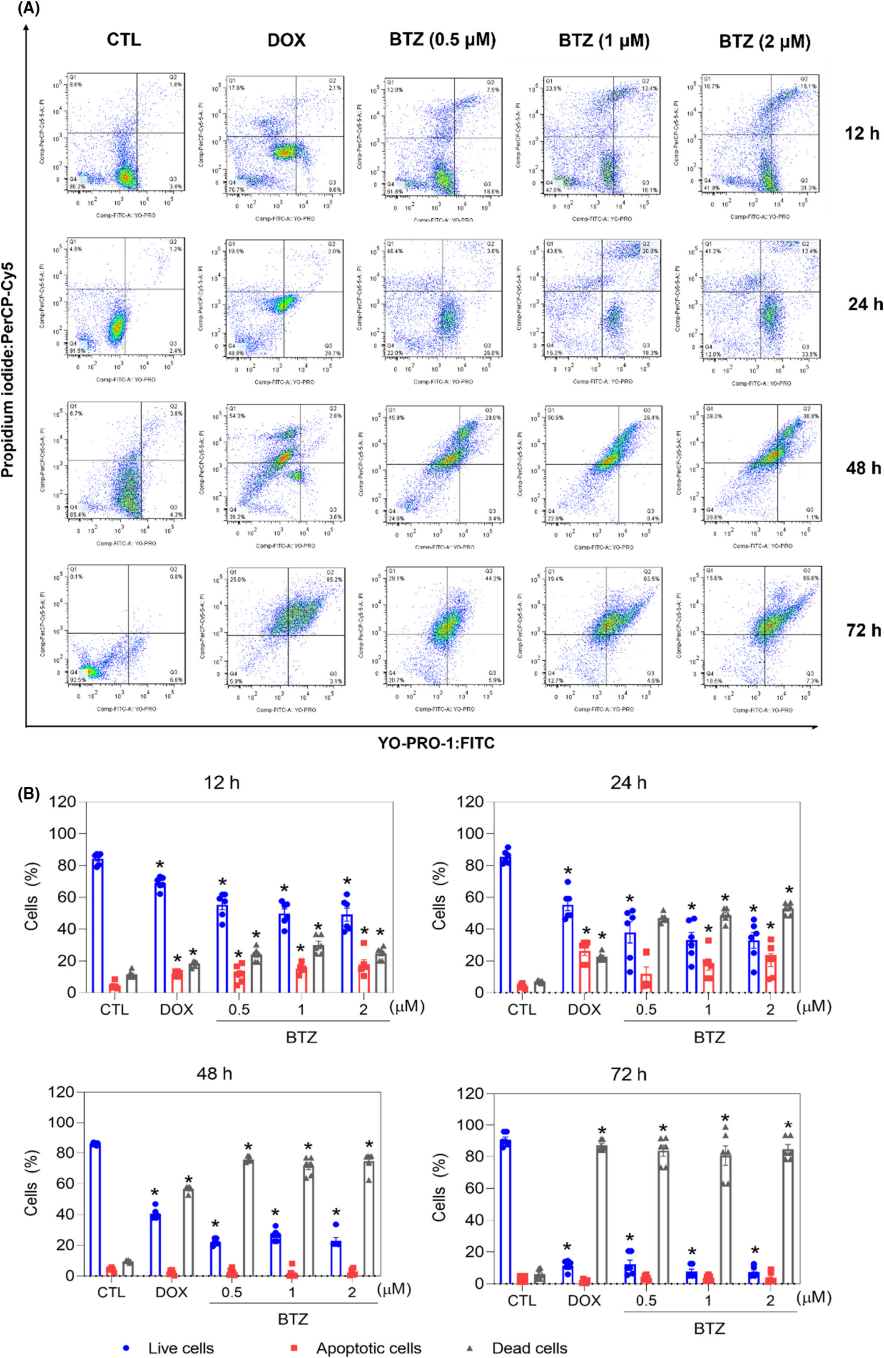
BTZ induces apoptotic cell death in KG‐1a cells. (A) Representative flow cytometry dot plots. (B) Apoptosis quantification in KG‐1a cells after 12, 24, 48 and 72 h of treatment with BTZ. Quantification of live (YO‐PRO‐1‐ and PI‐negative cells), apoptotic (YO‐PRO‐1‐positive cells) and dead (YO‐PRO‐1/PI double‐positive cells plus PI‐positive cells) KG‐1a cells. Vehicle (0.2% DMSO) was used as a negative control (CTL), and doxorubicin (DOX, 1 μM) was used as a positive control. The data are shown as the mean ± S.E.M. of three independent experiments carried out in duplicate. *p < 0.05 compared with CTL by one‐way ANOVA followed by Dunnett's multiple comparisons test.

Apoptosis induction was measured in BTZ‐treated KG‐1a cells using YO‐PRO‐1/PI staining. Significant induction of apoptosis was detected after 12 and 24 h of incubation, while nonspecific cell death was detected after 48 and 72 h of incubation (Figure 5). At concentrations of 0.5, 1 and 2 μM, BTZ‐induced apoptosis by 11.8%, 15.1% and 17.8%, respectively, after 12 h of incubation (compared to 4.4% observed in the control), while 11.8%, 17.8% and 20.5%, respectively, were detected after 24 h of incubation (compared to 4.0% detected in the control). BTZ treatment also led to nuclear condensation, as detected by the increase in side scatter (a flow cytometry parameter of cell complexity/granularity), and cell shrinkage, as quantified by a reduction in forward light scatter (a flow cytometry parameter of cell size). These morphological changes are features of apoptotic cells (Figure S3).

**FIGURE 6:**
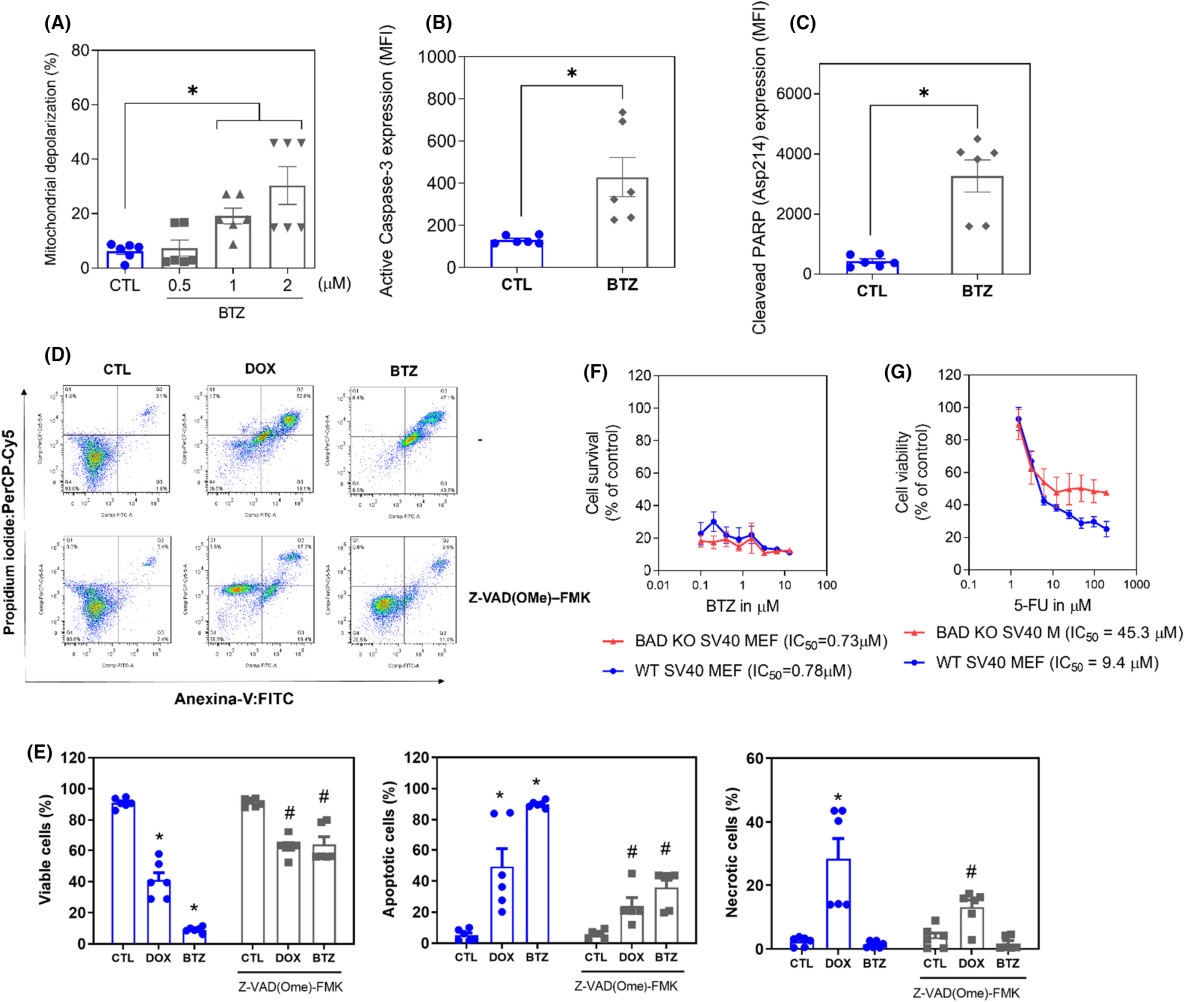
BTZ induces caspase‐mediated apoptotic cell death in KG‐1a cells. (A) Effect of BTZ on mitochondrial activity in KG‐1a cells. (B) Effect of BTZ on the levels of active caspase 3 and (C) cleaved PARP (Asp214) after 24 h of treatment in KG‐1a cells. (D and E) Effect of the pancaspase inhibitor Z‐VAD(OMe)‐FMK on BTZ‐induced apoptosis in KG‐1a cells. The cells were pretreated for 2 h with 50 μM Z‐VAD(OMe)‐FMK and then incubated with 2 μM BTZ for 48 h. (F and G) Survival curves of WT SV40 MEFs and BAD KO SV40 MEFs upon treatment with 5‐fluorouracil (5‐FU, used as a positive control) and BTZ. The curves were obtained from at least three independent experiments carried out in duplicate using the Alamar blue assay after 72 h of incubation. Vehicle (0.2% DMSO) was used as a negative control (CTL), and doxorubicin (DOX, 1 μM) was used as a positive control. The data are shown as the mean ± S.E.M. of three independent experiments carried out in duplicate. *p < 0.05 compared with CTL by Student's *t*‐test or one‐way ANOVA followed by Dunnett's multiple comparisons test. #*p* < 0.05 compared with the respective treatment without inhibitor by Student's *t*‐test. MFI, mean fluorescence intensity.

Next, increases in the loss of transmembrane mitochondrial potential (Figure 6A), active caspase‐3 (Figure 6B) and cleaved PARP‐(Asp 214) (Figure 6C) were also found in KG‐1a cells treated with BTZ. Moreover, BTZ‐induced cell death was partially prevented by pretreatment with the pancaspase inhibitor Z‐VAD‐(OMe)‐FMK (Figure 6D,E), indicating that BTZ induces caspase‐mediated apoptosis in KG‐1a cells. Accordingly, the proapoptotic genes *BAD* (RQ = 2.17) and *FAS* (RQ = 4.93) were upregulated by BTZ treatment in KG‐1a cells, as mentioned above. On the contrary, BTZ‐induced cytotoxicity occurred independently of BAD, as observed by comparing the cytotoxic effect of BTZ on BAD KO SV40 MEFs with that on parental control WT SV40 MEFs (Figure 6F,G), suggesting that BTZ could act through multiple cell death mechanisms.

### 3.5 BTZ causes oxidative stress‐mediated apoptosis in KG‐1a cells

**FIGURE 7:**
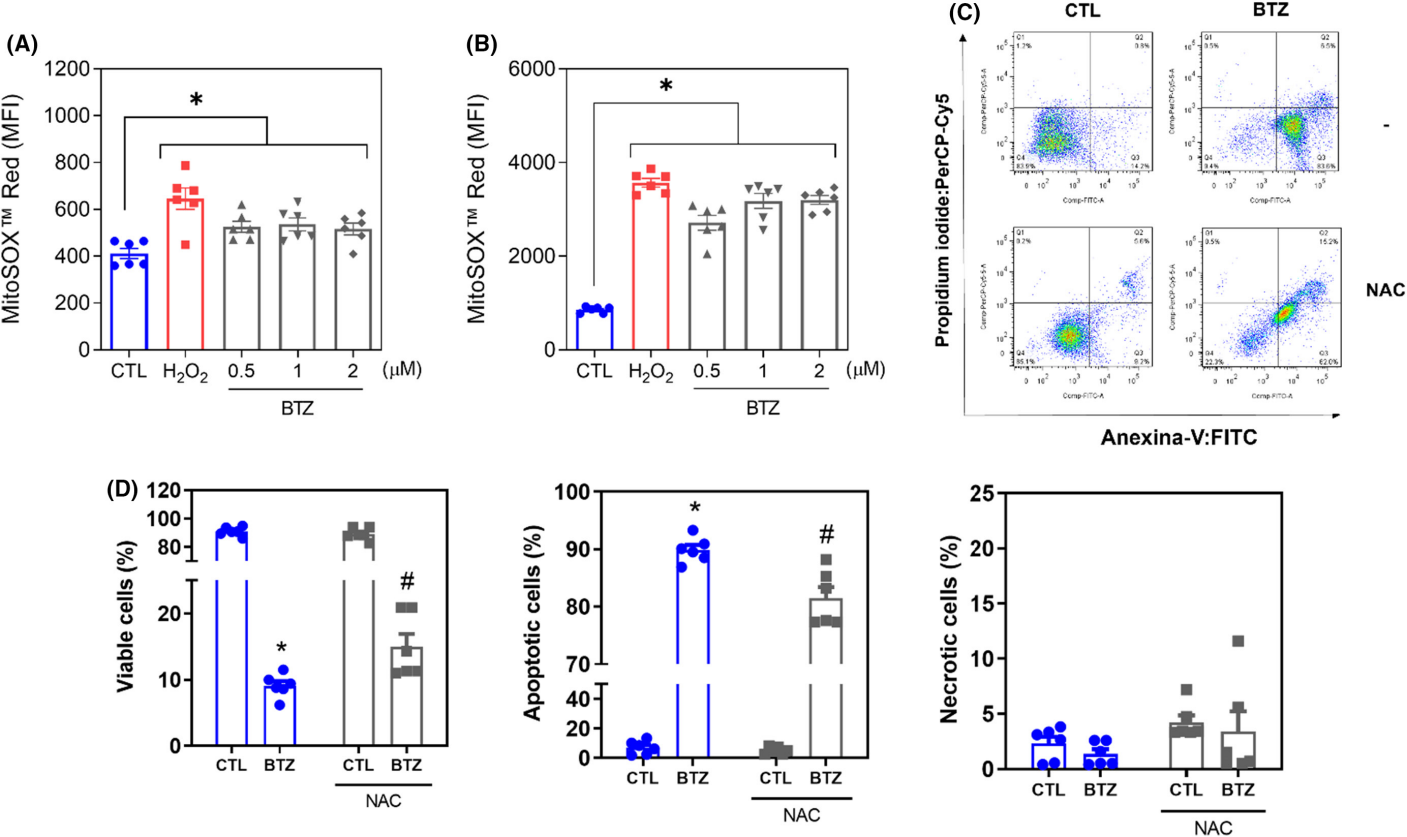
BTZ causes oxidative stress‐mediated apoptotic cell death in KG‐1a cells. Mitochondrial ROS in KG‐1a cells after 1 (A) and 24 (B) h of treatment with BTZ. (C and D) Effect of the antioxidant NAC on the apoptosis induced by BTZ in KG‐1a cells. The cells were pretreated for 2 h with 5 mM NAC and then incubated with BTZ at 2 μM for 48 h. Vehicle (0.2% DMSO) was used as a negative control (CTL), and hydrogen peroxide (H_2_O_2_, 100 μM) was used as a positive control. The data are shown as the mean ± S.E.M. of three independent experiments carried out in duplicate. *p < 0.05 compared with CTL by Student's *t*‐test or one‐way ANOVA followed by Dunnett's multiple comparisons test. #*p* < 0.05 compared with the respective treatment without inhibitor by Student's *t*‐test. MFI, mean fluorescence intensity.

MitoSOX™ Red was used to measure mitochondrial superoxide levels in KG‐1a cells after 1 and 24 h of incubation with BTZ. At concentrations of 0.5, 1 and 2 μM, BTZ‐enhanced mitochondrial superoxide levels in KG‐1a cells with MFIs of 526.3, 536.0 and 516.5 after 1 h of incubation (Figure 7A), while 2717, 3181 and 3203 were detected after 24 h of incubation (Figure 7B), respectively, compared to 411.8 and 857 detected in the negative control, suggesting the induction of oxidative stress by BTZ.

To assess whether the proapoptotic effect of BTZ was related to its induction of oxidative stress in KG‐1a cells, we used the antioxidant NAC. Interestingly, BTZ‐induced apoptosis was partially prevented by pretreatment with NAC (Figure 7C,D), indicating that BTZ induces, at least in part, oxidative stress‐mediated apoptosis in KG‐1a cells.

### 3.6 BTZ inhibits the development of KG‐1a cell xenografts in the bone marrow of NSG mice

**FIGURE 8:**
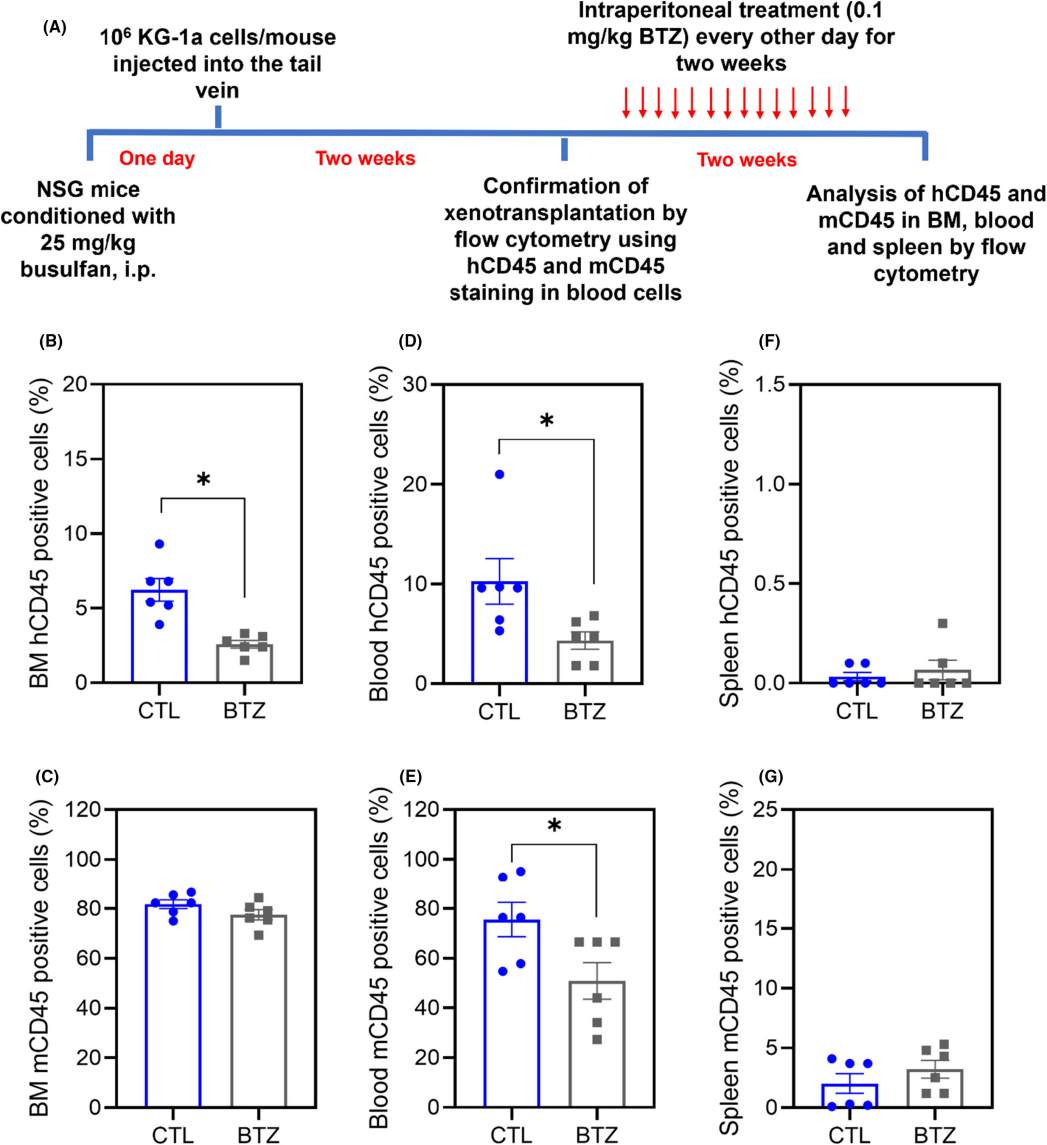
BTZ reduces the growth of KG‐1a cell xenografts in NSG mice. (A) Experimental design. Two weeks after the inoculation of KG‐1a cells, the mice were randomly divided into the BTZ (0.1 mg/kg) group and the control group (5% DMSO). hCD45‐positive cells were quantified by flow cytometry from (B) bone marrow (BM), (D) peripheral blood and (F) spleen. mCD45‐positive cells were quantified by flow cytometry from (C) BM, (E) peripheral blood and (G) spleen. The data are shown as the mean ± S.E.M. of 6 animals. *p < 0.05 compared with CTL by Student's *t*‐test.

The anti‐AML activity of BTZ was also investigated in a xenograft model of leukaemia, and the ability of BTZ to reduce the development of KG‐1a cell xenografts in the bone marrow of NSG mice was evaluated (Figure 8). At a dosage of 0.1 mg/kg every other day for 2 weeks, BTZ significantly reduced the percentage of hCD45‐positive cells in the bone marrow and peripheral blood of NSG mice engrafted with KG‐1a cells. Furthermore, BTZ reduced the percentage of mCD45‐positive cells in the peripheral blood but did not affect their percentage in the bone marrow or spleen.

Some toxicological aspects were also evaluated in BTZ‐treated NSG mice engrafted with KG‐1a cells (Figure S4). No statistically significant differences were found in body or organ (liver, lung, heart, spleen or kidney) weights.

Histological analyses were also performed on organs removed from BTZ‐treated animals (Figure S5). The livers showed altered histopathological changes, such as vascular hyperemia, hydropic degeneration of hepatocytes and mixed tissue inflammation. These changes ranged from mild to moderate in the control and BTZ groups. Focal areas with hepatocytes in coagulation necrosis were also observed. Parenchyma and portal system architectures ranged from preserved to partially altered in the control and BTZ groups. The architecture of the lung parenchyma was partially altered in all animals from the control and BTZ groups. This architectural alteration was mainly related to thickening of the alveolar septa with airspace atelectasis, which ranged from mild to severe. In addition, other histological changes observed were vascular hyperemia, edema and inflammation with predominant polymorphonuclear cell infiltration. In addition, focal areas of haemorrhage, hemosiderin deposits and fibrosis were observed in the animals' lungs.

Renal architecture was maintained in the control group, but in animals treated with BTZ it ranged maintained to partially altered. The histopathological alterations observed in this organ were moderate‐to‐intense vascular hyperemia and mild glomerular hyalinization with a decrease in the urinary space, which was more evident in the BTZ group. Furthermore, the animals showed focal areas of fibrosis, and renal cortex tubular cells with coagulation necrosis were observed in both groups. Discrete areas of haemorrhage were observed in animals treated with BTZ. The hearts of the control and BTZ groups did not show architectural or morphological alterations.

## 4 DISCUSSION

Herein, we report that BTZ has potent cytotoxic effects on both solid and haematological malignant cells and reduces the stemness features of the AML stem/progenitor cell line KG‐1a. Induction of caspase and oxidative stress‐mediated apoptosis were found in BTZ‐treated KG‐1a cells, along with NF‐κB signalling inhibition. Furthermore, BTZ suppressed the development of KG‐1a cell xenografts in the bone marrow of NSG mice.

BTZ was the first FDA‐approved proteasome inhibitor for clinical use and is currently an approved drug for the treatment of multiple myeloma and mantle cell lymphoma.^9^, ^25^ In patients with AML, although some clinical trials have reported that BTZ did not improve outcomes,^26^, ^27^ many clinical trials of BTZ combined with chemotherapy drugs are still ongoing or do not yet have published data.

In preclinical studies, BTZ‐induced cytotoxicity in primary AML blasts, including AML blasts with KMT2A/MLL rearrangements, and resulted in a reduction in colony formation.^12^, ^13^ BTZ caused DNA hypomethylation and suppressed gene transcription in AML cells by interfering with Sp1/NF‐κB‐dependent DNA methyltransferase activity.^28^ Furthermore, BTZ inhibited Sp1/NFB gene transactivation, resulting in a decrease in KIT expression in AML cells.^29^

Although many studies have evaluated the effect of BTZ on solid and haematological malignant cells, only a few studies have focused on cancer/leukaemia stem cells. As mentioned above, BTZ has been previously demonstrated to inhibit the stemness properties of KMT2A/MLL‐rearranged AML by NF‐ĸB‐dependent inhibition of CDK6.^13^ On the contrary, Bosman et al.^12^ reported that BTZ fails to inhibit NF‐κB activity in AML stem/progenitor cells, although it is effective in these cells. In this study, we demonstrated that BTZ reduces the stemness features of AML stem‐like cells in the KG‐1a cell line by inhibiting NF‐ĸB and inducing oxidative stress. Interestingly, corroborating our findings, both the NF‐ĸB signalling pathway and the induction of oxidative stress are described as selective targets for eliminating LSC AML.^7^, ^8^ Treatment of KG‐1a cells with BTZ also resulted in downregulation of the hedgehog signalling receptor gene *PTCH1* and the epithelial–mesenchymal transition marker genes *SNAI3* and *TWIST1*, indicating that other mechanisms could also participate in the effects of BTZ in AML cells.

Induction of oxidative stress was also found in BTZ‐treated KG‐1a cells, and BTZ‐induced apoptosis was partially dependent on oxidative stress in this AML stem/progenitor cell line. Rushworth et al.^30^ previously demonstrated that BTZ induces cell death by inducing oxidative stress and that AML cells with high basal nuclear levels of Nrf2 are less sensitive to BTZ cytotoxicity. Moreover, Huang et al.^31^ reported that BTZ causes oxidative stress‐mediated apoptosis in OCI‐AML3 cells.

The anti‐AML activity of BTZ was also evaluated in an in vivo model using NSG mice inoculated with KG‐1a cells. In this model, we used immunosuppressed mice with AML cells engrafted in their bone marrow. We found that this drug is able to reduce leukaemic cells in the bone marrow and blood of animals, indicating that BTZ has the ability to eliminate AML cells in an in vivo model. The animals were treated with 0.1 mg/kg BTZ every other day for 2 weeks, and no significant toxicity was detected. Histological evaluation of organs from BTZ‐treated mice revealed only reversible changes. Previously, BTZ surprised the proliferation of leukaemic cells in NSG mice inoculated with KMT2A/MLL‐rearranged AML cells.^13^ Taken together, these data indicate that the anti‐LSC potential of BTZ may be an important strategy for the treatment of AML.

## AUTHOR CONTRIBUTIONS

**Rafaela G. A. Costa:** Conceptualization (equal); formal analysis (equal); investigation (equal); visualization (equal); writing – review and editing (equal). **Maiara de S. Oliveira:** Formal analysis (equal); investigation (equal); visualization (equal); writing – review and editing (equal). **Ana Carolina B. da C. Rodrigues:** Formal analysis (equal); investigation (equal); visualization (equal); writing – review and editing (equal). **Suellen L. R. Silva:** Formal analysis (equal); investigation (equal); visualization (equal); writing – review and editing (equal). **Ingrid R. S. B. Dias:** Formal analysis (equal); investigation (equal); visualization (equal); writing – review and editing (equal). **Milena B. P. Soares:** Funding acquisition (equal); project administration (equal); supervision (equal); visualization (equal); writing – review and editing (equal). **Ludmila de Faro Valverde:** Formal analysis (equal); investigation (equal); methodology (equal); visualization (equal); writing – review and editing (equal). **Clarissa Araujo Gurgel Rocha:** Formal analysis (equal); funding acquisition (equal); investigation (equal); methodology (equal); writing – review and editing (equal). **Rosane Borges Dias:** Conceptualization (equal); formal analysis (equal); investigation (equal); supervision (equal); visualization (equal); writing – review and editing (equal). **Daniel P. Bezerra:** Conceptualization (lead); data curation (equal); funding acquisition (lead); project administration (lead); supervision (lead); writing – original draft (lead).

## FUNDING INFORMATION

This work received financial support and fellowships from the Brazilian agencies Coordenação de Aperfeiçoamento de Pessoal de Nível Superior (CAPES), Conselho Nacional de Desenvolvimento Científico e Tecnológico (CNPq), Fundação Oswaldo Cruz (INOVA‐FIOCRUZ) and Fundação de Amparo à Pesquisa do Estado da Bahia (FAPESB, Brazil).

## CONFLICT OF INTEREST STATEMENT

The authors have no conflicts of interest.

## INFORMED CONSENT

All subjects provided signed informed consent prior to the use of these human specimens for research purposes.

## ANIMAL STUDIES

The experimental protocol was approved by a local animal ethics committee (#16/2018).

## Supporting information


Appendix S1.

## Data Availability

The data that support the findings of this study are available from the corresponding author upon reasonable request.
