# Supplementary material for: Bortezomib suppresses acute myelogenous leukaemia stem‐like KG‐1a cells via NF‐κB inhibition and the induction of oxidative stress
Source: J Cell Mol Med. 2024 Apr 23;28(8):e18333. doi: 10.1111/jcmm.18333 (PMC11037403; doi:10.1111/jcmm.18333)
Supplement: Supplementary file 1 — Appendix S1. [file JCMM-28-e18333-s001.pdf]

Supplemental Figure 1. BTZ reduces the viability of KG-1a cells. Trypan blue exclusion assay after (**A**) 12, (**B**) 24, (**C**) 48 and (**D**) 72 h of treatment of KG-1a cells with BTZ. Vehicle (0.2% DMSO) was used as a negative control (CTL), and doxorubicin (DOX, 1 μM) was used as a positive control. The data are expressed as the mean ± S.E.M. of three independent experiments carried out in duplicate. * $p < 0.05$ compared with CTL by one-way ANOVA followed by Dunnett's multiple comparisons test.

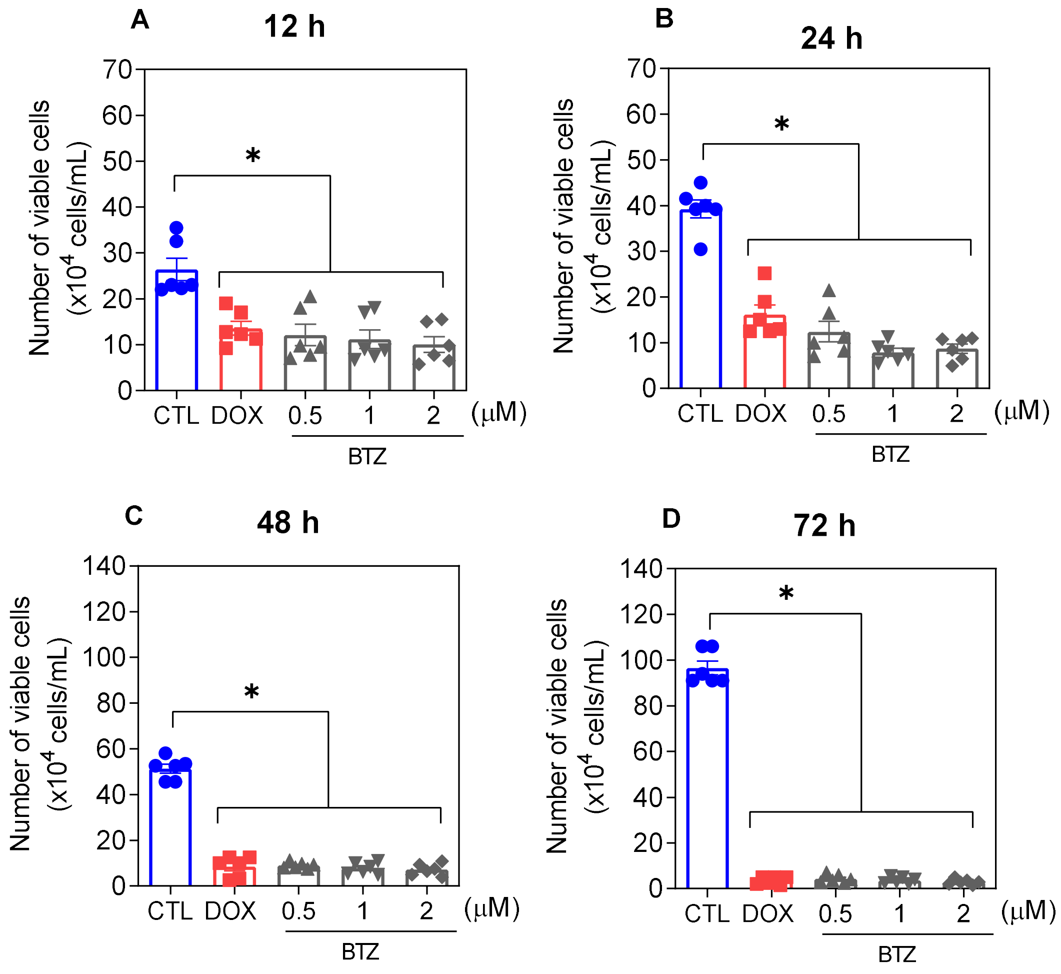

Supplemental Figure 2. BTZ did not change the expression of CD11b in KG-1a cells. (**A**) Immunophenotypic analysis of the myeloid differentiation marker CD11b in KG-1a cells treated with 2 μM BTZ after 24 h of incubation. The vehicle (0.2% DMSO) was used as a negative control (CTL). The data are expressed as the mean ± S.E.M. of three independent experiments carried out in duplicate. (**B**) Representative flow cytometry histogram of PBMCs stained with the anti-CD11b antibody, which was used as a positive control.

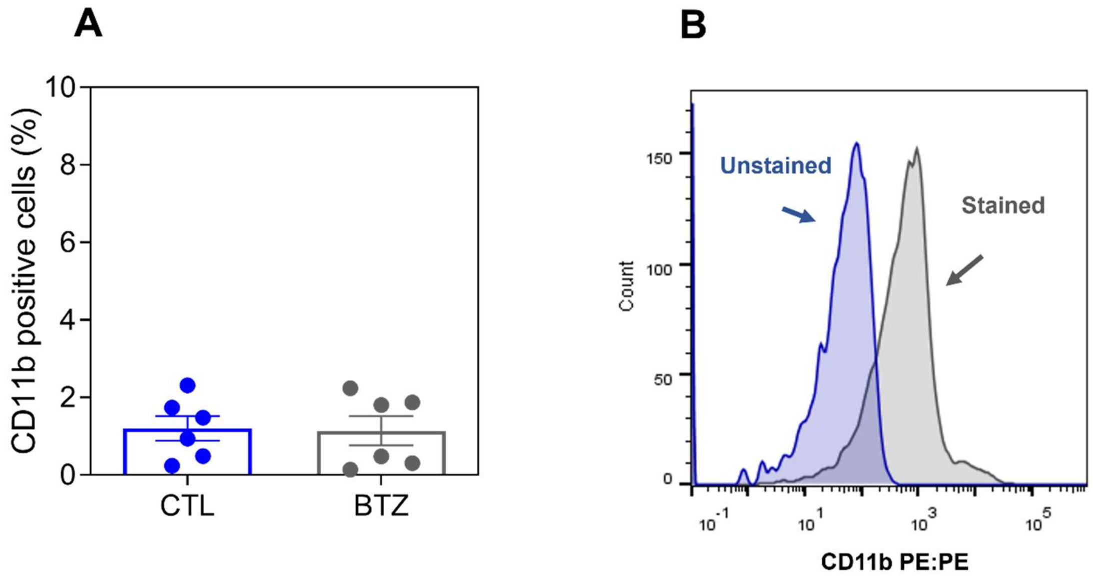

.

Supplemental Figure 3. BTZ affects the morphology of KG-1a cells. The light-scattering features (forward scatter – FSC and side scatter – SSC) were detected by flow cytometry after 12 (**A, B** and **C**), 24 (**A, D** and **E**), 48 (**A, F** and **G**) and 72 (**A, H** and **I**) h of incubation. The vehicle (0.2% DMSO) was used as a negative control (CTL), and doxorubicin (DOX, 1 μM) was used as a positive control. The data are expressed as the mean ± S.E.M. of three independent experiments carried out in duplicate. * $P < 0.05$ compared to CTL by one-way ANOVA followed by Dunnett's multiple comparisons test.

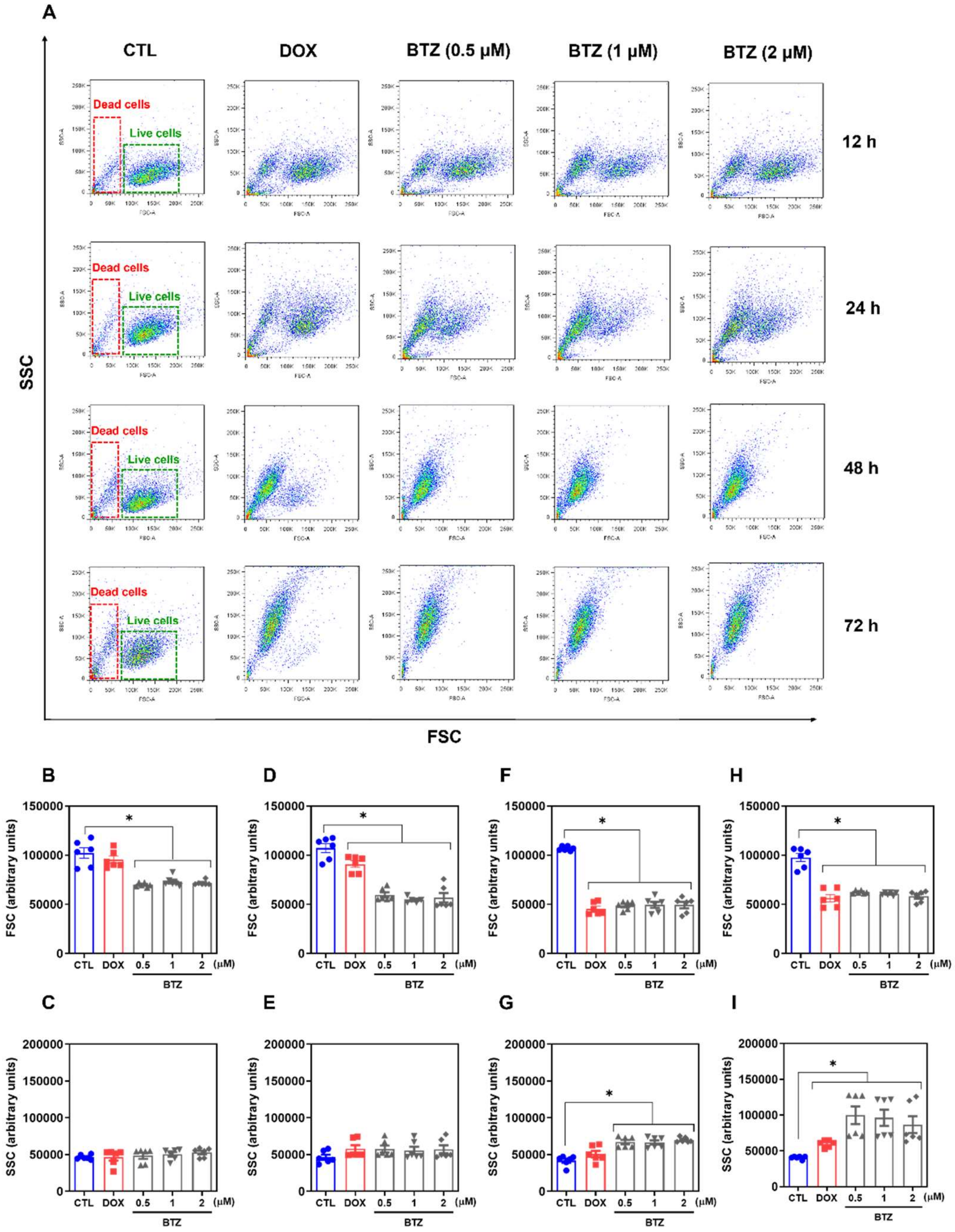

Supplemental Figure 4. BTZ treatment did not affect the body weight or relative organ weight of NSG mice with KG-1a cell xenografts. The negative control (CTL) was treated with the vehicle (5% DMSO) used for diluting BTZ. The treatment (0.1 mg/kg BTZ) was injected into the mice intraperitoneally every other day for 2 weeks. The data are expressed as the mean ± S.E.M. from 6 animals.

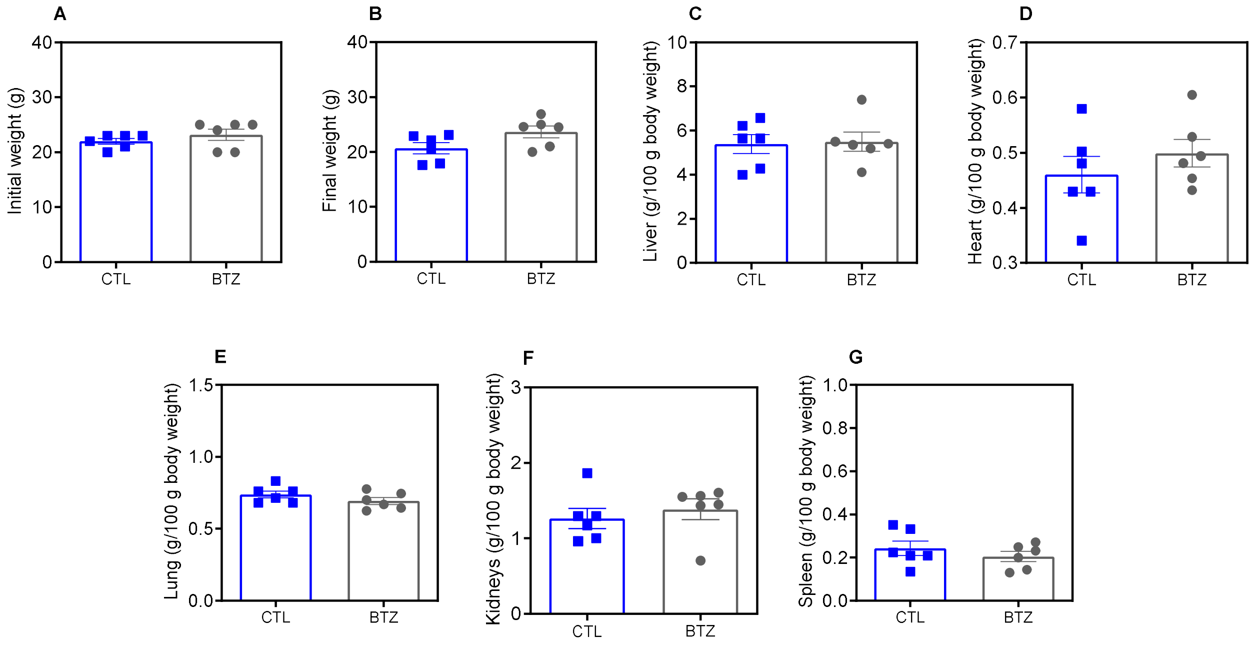

Supplemental Figure 5. Representative photomicrographs of the kidneys, lungs and livers of NSG mice with AML KG-1a cell xenografts. The negative control (CTL) was treated with the vehicle (5% DMSO) used for diluting BTZ. The treatment (0.1 mg/kg BTZ) was injected into the mice intraperitoneally every other day for 2 weeks. Scale bar = 100 μm.

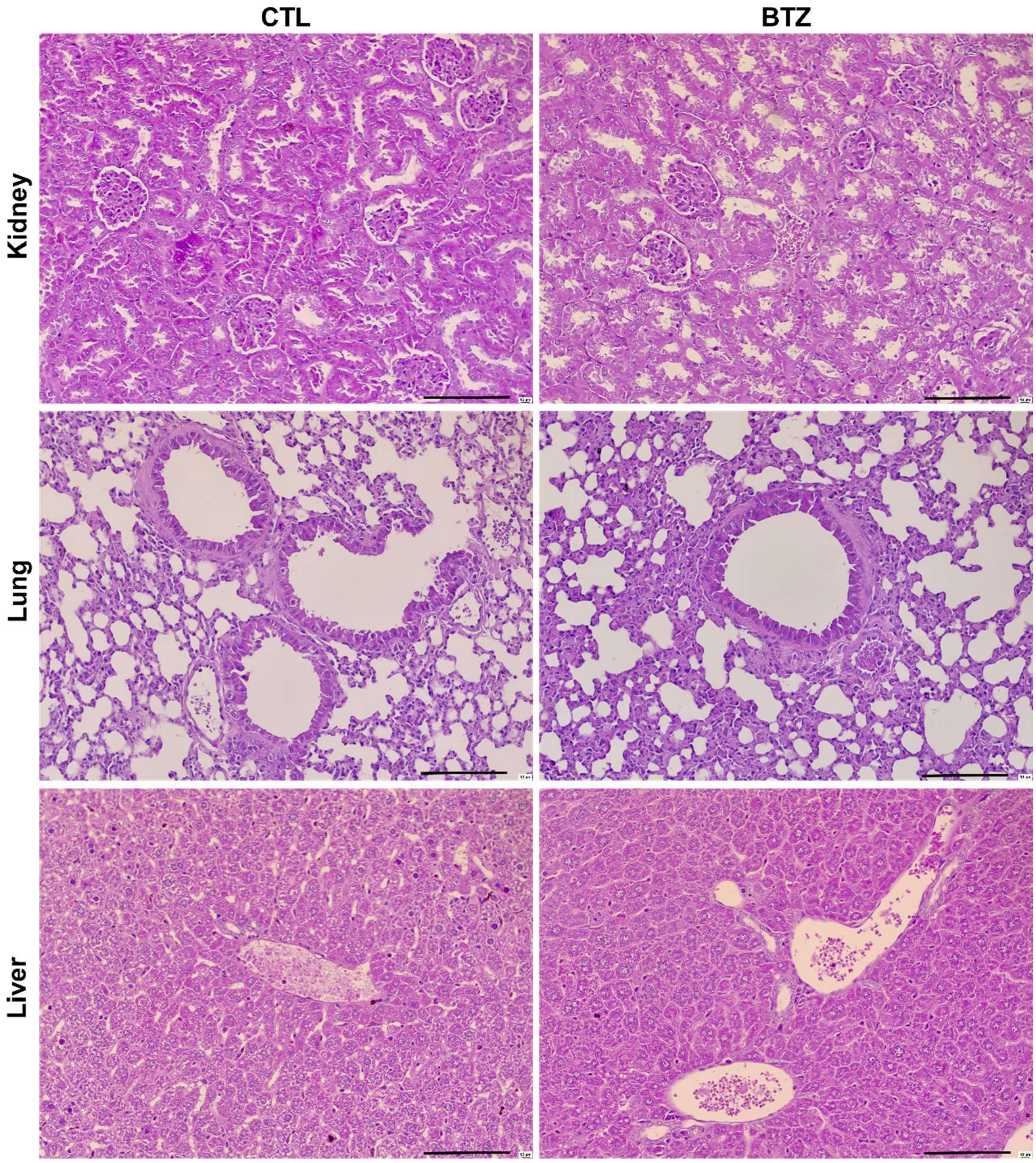

Supplemental Table 1. List of cells used

Cells	Histological type	Species	Source[a,b]
Haematological cancer cell lines			
Jurkat	T-cell lymphoid leukaemia	human	ATCC
KG-1a	acute myelogenous leukaemia	human	ATCC
NB4	acute promyelocytic leukaemia	human	ATCC
HL-60	acute promyelocytic leukaemia	human	ATCC
K-562	chronic myelogenous leukaemia	human	ATCC
THP-1	monocytic leukaemia	human	ATCC
Solid cancer cell lines			
MDA-MB-231	breast carcinoma	human	BCRJ
MCF-7	breast adenocarcinoma	human	ATCC
4T1	breast carcinoma	mouse	ATCC
HCT116	colorectal carcinoma	human	ATCC
B16-F10	melanoma	mouse	ATCC
A-375	melanoma	human	BCRJ
HepG2	hepatocellular carcinoma	human	ATCC
OVCAR-3	ovarian carcinoma	human	BCRJ
U-87 MG	glioblastoma	human	BCRJ
A549	lung adenocarcinoma	human	BCRJ
PANC-1	pancreas ductal adenocarcinoma	human	BCRJ
DU 145	prostate carcinoma	human	BCRJ
HSC-3	oral squamous cell carcinoma	human	ATCC

CAL 27	oral squamous cell carcinoma	human	ATCC
SSC-4	oral squamous cell carcinoma	human	ATCC
SCC-9	oral squamous cell carcinoma	human	ATCC
SSC-25	oral squamous cell carcinoma	human	ATCC
Noncancer cell lines			
MRC-5	lung fibroblast	human	ATCC
BJ	foreskin fibroblast	human	ATCC
Mutant and its parental cell lines			
BAD KO SV40 MEF	immortalized mouse embryonic fibroblasts with the BAD gene knocked out	mouse	ATCC
WT SV40 MEF	wild-type immortalized embryonic fibroblasts	mouse	ATCC
Primary cells			
PBMC	health peripheral blood mononuclear cells	human	primary cell culture

[a]ATCC denotes the American Type Culture Collection (USA). [b]Primary cell culture of PBMCs was obtained from human peripheral blood from healthy donors by a standard Ficoll density protocol. Then, the PBMCs were resuspended in RPMI 1640 or DMEM-F12 supplemented with 20% FBS and 1% antibiotics. The cells were plated at 5×10^5 cells/well. Concanavalin A (10 µg/mL, Sigma–Aldrich) was used as a mitogen to trigger cell division in T lymphocytes and was added at the beginning of the culture. The Research Ethics Committee of the Oswaldo Cruz Foundation (Salvador, Bahia, Brazil) approved the protocol (CAAE 16220713.2.0000.0040). All donors signed the Free and Informed Consent Form.

Supplemental Table 2. Antibodies used

Antibody	Fluorochrome	Reactivity	Clone	Catalog number	Manufacturer
Active caspase-3	FITC	Human/Mouse	C92-605	559341	BD Pharmingen™
Cleaved PARP (Asp214)	PE	Human	F21-852	552933	BD Pharmingen™
NF-κB p65	PE	Human	14G10A21	653004	BioLegend
CD11b	PE	Human	Clone D12	347557	BD™
CD13	PE-CF594	Human	WM15	562491	BD Horizon™
CD33	BV510	Human	WM53	563257	BD Horizon™
CD34	PE	Human	8G12	348057	BD™
CD38	BV421	Human	HIT2	562444	BD Horizon™
CD123	BV605	Human	7G3	564197	BD Horizon™
CD45 (hCD45)	PE	Human	2D1	368509	BioLegend
CD45 (mCD45)	FITC	Mouse	30-F11	103107	BioLegend

IgG1, κ Isotype Control	PE	-	MOPC-21	556650	BD Pharmingen™

Supplemental Table 3. Cytotoxicity of BTZ

**Cells**	**IC_{50} and 95% CI (µM)**	
	**BTZ**	**DOX**
Haematological cancer cells		
Jurkat	0.05	0.04
	0.01 – 1.86	0.02 – 0.11
KG-1a	0.06	0.54
	0.01 – 0.90	0.23 – 1.25
NB4	0.11	0.13
	0.04 – 0.33	0.08 – 0.21
HL-60	0.17	0.17
	0.03 – 1.10	0.10 – 0.28
K-562	0.31	1.35
	0.09 – 1.01	0.75 – 2.43
THP-1	0.33	0.23
	0.10 – 1.06	0.14 – 0.38
Solid cancer cells		
MCF-7	1.39	1.33
	0.44 – 4.41	0.85 – 2.08
MDA-MB-231	1.48	2.00
	0,52 – 2.27	1.14 – 3.50
4T1	0.78	1.53
	0.36 – 2.58	1.09 – 2.17
HCT116	0.31	0.08

	0.06 – 1.75	0.04 – 0.15
B16-F10	0.03	0.10
	0.01 – 0.39	0.06 – 0.16
A375	0.01	0.46
	0.01 – 0.12	0.33 – 0.65
HepG2	0.16	0.28
	0.01 – 1.81	0.16 – 0.48
OVCAR-3	0.05	1.84
	0.01 – 0.95	1.13 – 3.00
U-87 MG	1.67	0.48
	0.67 – 4.18	0.24 – 0.98
A549	0.14	2.54
	0.12 – 0.16	1.96 – 3.30
PANC-1	0.02	4.36
	0.01 – 0.14	2.99 – 6.34
DU 145	0.09	1.35
	0.02 – 0.56	0.52 – 3.50
HSC-3	0.02	0.48
	0.01 – 0.26	0.36 – 0.63
CAL27	0.08	0.27
	0.04 – 0.16	0.166 – 0.47
SCC4	0.18	0.29
	0.04 – 0.74	0.21 – 0.40
SSC-9	1.97	1.14
	0.98 – 3.96	0.64 – 2.01
SSC-25	1.07	2.99

	0.39 – 2.95	1.82 – 4.91
Noncancerous cells		
MRC-5	0.58	1.44
	0.14 – 2.51	0.90 – 2.30
BJ	0.21	2.65
	0.04 – 1.10	1.51 – 4.66
PBMC	0.51	1.33
	0.18 – 1.47	0.91 – 1.94

These data were obtained by nonlinear regression from three independent experiments performed in duplicate. Doxorubicin (DOX) was used as a positive control.

Supplemental Table 4. Selectivity indices obtained

Cancer cells	Noncancerous cells					
	MRC-5		BJ		PBMC	
	BTZ	DOX	BTZ	DOX	BTZ	DOX
Haematological cancer cells						
Jurkat	11.6	36.0	4.2	66.3	10.2	33.3
KG-1a	9.7	2.7	3.5	4.9	8.5	2.5
NB4	5.3	11.1	1.9	20.4	4.6	10.2
HL-60	3.4	8.5	1.2	15.6	3.0	7.8
K-562	1.9	1.1	0.7	2.0	1.6	1.0
THP-1	1.8	6.3	0.6	11.5	1.5	5.8
Solid cancer cells						
MCF-7	0.4	1.1	0.2	2.0	0.4	1.0
MDA-MB-231	0.4	0.7	0.1	1.3	0.3	0.7
4T1	0.7	0.9	0.3	1.7	0.7	0.9
HCT116	1.9	18.0	0.7	33.1	1.6	16.6
B16-F10	19.3	14.4	7.0	26.5	17.0	13.3
A375	58.0	3.1	21.0	5.8	51.0	2.9
HepG2	3.6	5.1	1.3	9.5	3.2	4.8
OVCAR-3	11.6	0.8	4.2	1.4	10.2	0.7
U-87 MG	0.3	3.0	0.1	5.5	0.3	2.8
A549	4.1	0.6	1.5	1.0	3.6	0.5
PANC-1	29.0	0.3	10.5	0.6	25.5	0.3

DU 145	6.4	1.1	2.3	2.0	5.7	1.0
HSC-3	29.0	3.0	10.5	5.5	25.5	2.8
CAL27	7.3	5.3	2.6	9.8	6.4	4.9
SCC4	3.2	5.0	1.2	9.1	2.8	4.6
SSC-9	0.3	1.3	0.1	2.3	0.3	1.2
SSC-25	0.5	0.5	0.2	0.9	0.5	0.4

The data were calculated using the following formula: Selectivity indices = IC_{50} [noncancerous cells]/IC_{50} [cancer cells].

Supplemental Table 5. The effect of BTZ on gene expression in KG-1a cells

Function/Assay ID	Gene Symbol	Gene Name	RQ	
			CTL	BTZ
**NFkB pathway**				
Hs00765730_m1	NFKB1	nuclear factor kappa B subunit 1	1.00	0.78
Hs00174517_m1	NFKB2	nuclear factor kappa B subunit 2	1.00	1.83
Hs00153283_m1	NFKBIA	NFKB inhibitor alpha	1.00	1.90
Hs00182115_m1	NFKBIB	NFKB inhibitor beta	1.00	2.21
Hs00153294_m1	RELA	RELA proto-oncogene, NF-kB subunit	1.00	2.91
Hs00232399_m1	RELB	RELB proto-oncogene, NF-kB subunit	1.00	7.31
**WNT/β-catenin pathway**				
Hs00181051_m1	APC	APC, WNT signalling pathway regulator	1.00	2.17
Hs00793391_m1	CSNK1A1	casein kinase 1 alpha 1	1.00	1.35
Hs00170025_m1	CTNNB1	catenin beta 1	1.00	1.38
Hs00275656_m1	GSK3B	glycogen synthase kinase 3 beta	1.00	1.63
Hs00228741_m1	WNT10A	Wnt family member 10A	n.d.	n.d.
Hs00559664_m1	WNT10B	Wnt family member 10B	1.00	1.25
Hs00257131_m1	WNT2B	Wnt family member 2B	n.d.	n.d.
Hs00362452_m1	WNT6	Wnt family member 6	n.d.	n.d.
**Hedgehog pathway**				

Hs00368306_m1	DHH	desert hedgehog	n.d.	n.d.
Hs00171790_m1	GLI1	GLI family zinc finger 1	1.00	1.08
Hs00257977_m1	GLI2	GLI family zinc finger 2	n.d.	n.d.
Hs00181117_m1	PTCH1	patched 1	1.00	0.19
Hs00179843_m1	SHH	sonic hedgehog	n.d.	n.d.
Hs00170665_m1	SMO	smoothened, frizzled class receptor	n.d.	n.d.
Hs00171981_m1	SUFU	SUFU negative regulator of hedgehog signalling	1.00	1.63
**NOTCH pathway**				
Hs00194509_m1	DLL1	delta like canonical Notch ligand 1	1.00	n.d.
Hs01085096_m1	DLL3	delta like canonical Notch ligand 3	1.00	n.d.
Hs00164982_m1	JAG1	jagged 1	1.00	2.79
Hs00171432_m1	JAG2	jagged 2	1.00	7.47
Hs01062014_m1	NOTCH1	notch 1	1.00	0.83
Hs01050702_m1	NOTCH2	notch 2	1.00	0.94
**EGFR pathway**				
Hs01099999_m1	EGF	epidermal growth factor	1.00	26.61
Hs01076078_m1	EGFR	epidermal growth factor receptor	n.d.	n.d.
Hs00364282_m1	KRAS	KRAS proto-oncogene, GTPase	1.00	1.09
Hs01046830_m1	MAPK1	mitogen-activated protein kinase 1	1.00	1.42

Hs00234119_m1	RAF1	Raf-1 proto-oncogene, serine/threonine kinase	1.00	1.57
Hs00269660_s1	RHOB	ras homolog family member B	n.d.	n.d.
**JAK/STAT pathway**				
Hs01026983_m1	JAK1	Janus kinase 1	1.00	1.23
Hs01078136_m1	JAK2	Janus kinase 2	1.00	0.69
Hs00169663_m1	JAK3	Janus kinase 3	n.d.	n.d.
Hs01013989_m1	STAT1	signal transducer and activator of transcription 1	1.00	2.45
Hs00374280_m1	STAT3	signal transducer and activator of transcription 3	1.00	3.09
Hs00273500_m1	STAT5B	signal transducer and activator of transcription 5B	1.00	2.53
Hs00598625_m1	STAT6	signal transducer and activator of transcription 6	1.00	1.56
**PI3K/AKT/MTOR pathway**				
Hs00178289_m1	AKT1	AKT serine/threonine kinase 1	1.00	1.85
Hs01086102_m1	AKT2	AKT serine/threonine kinase 2	1.00	2.21
Hs00234508_m1	MTOR	mechanistic target of rapamycin	1.00	2.43
Hs00904054_m1	PIK3C2A	phosphatidylinositol-4-phosphate 3-kinase catalytic subunit type 2 alpha	1.00	2.02

Hs00176908_m1	PIK3C3	phosphatidylinositol 3-kinase catalytic subunit type 3	1.00	1.59
Hs00907957_m1	PIK3CA	phosphatidylinositol-4,5-bisphosphate 3-kinase catalytic subunit alpha	1.00	2.00
Hs02621230_s1	PTEN	phosphatase and tensin homolog	1.00	0.73
**TGF-beta/SMAD pathway**				
Hs01054576_m1	FOXO1	forkhead box O1	1.00	0.55
Hs00183425_m1	SMAD2	SMAD family member 2	1.00	2.17
Hs00929647_m1	SMAD4	SMAD family member 4	1.00	1.14
Hs00178696_m1	SMAD7	SMAD family member 7	1.00	3.07
Hs00998133_m1	TGFB1	transforming growth factor beta 1	1.00	1.38
Hs00234244_m1	TGFB2	transforming growth factor beta 2	1.00	n.d.
Hs01086000_m1	TGFB3	transforming growth factor beta 3	1.00	n.d.
**PPAR pathway**				
Hs00947536_m1	PPARA	peroxisome proliferator activated receptor alpha	1.00	1.86
Hs04187066_g1	PPARD	peroxisome proliferator activated receptor delta	1.00	2.00
Hs01115513_m1	PPARG	peroxisome proliferator activated receptor gamma	1.00	9.98
Hs01016719_m1	PPARGC1A	PPARG coactivator 1 alpha	n.d.	n.d.
Hs00991677_m1	PPARGC1B	PPARG coactivator 1 beta	1.00	0.76

**Oxidative stress**				
Hs00943350_g1	GSTP1	glutathione S-transferase pi 1	1.00	2.10
Hs00178247_m1	OXSR1	oxidative stress responsive 1	1.00	0.95
Hs00167309_m1	SOD2	superoxide dismutase 2, mitochondrial	1.00	1.48
Hs01555214_g1	TXN	thioredoxin	1.00	1.70
Hs00917067_m1	TXNRD1	thioredoxin reductase 1	1.00	6.26
**Apoptosis**				
Hs00559441_m1	APAF1	apoptotic peptidase activating factor 1	1.00	1.74
Hs00188930_m1	BAD	BCL2 associated agonist of cell death	1.00	2.17
Hs00180269_m1	BAX	BCL2 associated X, apoptosis regulator	1.00	1.40
Hs99999018_m1	BCL2	BCL2, apoptosis regulator	1.00	0.62
Hs00609632_m1	BID	BH3 interacting domain death agonist	1.00	1.43
Hs00234387_m1	CASP3	caspase 3	1.00	1.37
Hs00169152_m1	CASP7	caspase 7	1.00	1.01
Hs00219876_m1	DIABLO	diablo IAP-binding mitochondrial protein	1.00	1.39
Hs00538709_m1	FADD	Fas associated via death domain	1.00	1.03
Hs00531110_m1	FAS	Fas cell surface death receptor	1.00	4.93

Hs00242302_m1	PARP1	poly(ADP-ribose) polymerase 1	1.00	0.51
**Autophagy**				
Hs01047860_g1	ATG12	autophagy related 12	1.00	1.63
Hs00223937_m1	ATG3	autophagy related 3	1.00	0.56
Hs00169468_m1	ATG5	autophagy related 5	1.00	1.47
Hs00186838_m1	BECN1	beclin 1	1.00	0.67
Hs01076567_g1	MAP1LC3A	microtubule associated protein 1 light chain 3 alpha	1.00	n.d.
Hs00797944_s1	MAP1LC3B	microtubule associated protein 1 light chain 3 beta	1.00	5.53
**Necroptosis**				
Hs00169407_m1	RIPK1	receptor interacting serine/threonine kinase 1	1.00	1.96
Hs01572686_m1	RIPK2	receptor interacting serine/threonine kinase 2	1.00	1.93
Hs01011177_g1	RIPK3	receptor interacting serine/threonine kinase 3	1.00	1.96
**Epithelial–mesenchymal transition**				
Hs00170423_m1	CDH1	cadherin 1	n.d.	n.d.
Hs00195591_m1	SNAI1	snail family transcriptional repressor 1	1.00	11.86
Hs00950344_m1	SNAI2	snail family transcriptional repressor 2	n.d.	n.d.

Hs01018996_m1	SNAI3	snail family transcriptional repressor 3	1.00	0.25
Hs00361186_m1	TWIST1	twist family bHLH transcription factor 1	1.00	0.16
Hs00185584_m1	VIM	vimentin	1.00	5.23
Hs00232783_m1	ZEB1	zinc finger E-box binding homeobox 1	1.00	1.2
Hs00207691_m1	ZEB2	zinc finger E-box binding homeobox 2	1.00	12.34

KG-1a cells were treated with 2 μM BTZ for 12 h. The negative control (CTL) was treated with the vehicle (0.2% DMSO) used for diluting BTZ. After treatment, total RNA was isolated and reverse transcribed. Gene expression was detected using a TaqMan® array plate 96 plus fast (#4413256). The GUSB, HPRT1 and GAPDH genes were used as endogenous genes for normalization. The values represent the relative quantitation (RQ) compared with the calibrator (cells treated with the negative control). The genes were considered to be upregulated if RQ ≥ 2 and downregulated if RQ ≤ 0.5. N.d. Not determined.
